# Drug repurposing of fostamatinib against cancer via potential cytotoxicity and immune checkpoint regulation

**DOI:** 10.3389/fimmu.2025.1602189

**Published:** 2025-05-29

**Authors:** Maoqiong Hu, Renyi Yin, Kaifeng Deng, Ning Xu

**Affiliations:** ^1^ Department of Medical Record Statistics, Wuhan No.1 Hospital, Wuhan, China; ^2^ Medical Science Laboratory, The Fourth Affiliated Hospital of Guangxi Medical University, Liuzhou, China; ^3^ Department of Clinical Medicine, The Fifth Affiliated Hospital of Guangxi Medical University, Nanning, China

**Keywords:** fostamatinib, cytotoxicity, immune checkpoint, network pharmacology, drug repurposing

## Abstract

Acute myeloid leukemia (AML), originating from myeloid hematopoietic stem/progenitor cells, is a malignant hematological disorder. Resistance to current treatments, especially in FLT3-ITD AML cases, urgently demands the development of novel therapeutics. In this study, we pinpointed fostamatinib, an orally delivered small molecule SYK inhibitor for chronic immune thrombocytopenia (ITP), as a promising candidate for drug repurposing. It effectively inhibited FLT3-ITD+ AML cell proliferation and induced leukemic cell apoptosis. Network pharmacology analysis further deciphered the associated pharmacological mechanism related to the PI3K-AKT signaling pathway. Moreover, fostamatinib downregulated the expression of immune checkpoints such as PD-L1 and CD47. Overall, this study provided a conceptual foundation for evaluating the advantages of drug repurposing in AML drug development.

## Introduction

Acute myeloid leukemia (AML) is a malignant hematological disease caused by myeloid hematopoietic stem/progenitor cells. Currently, the most prevalent genetic variation is the fms-like tyrosine kinase 3 (flt3) mutation. Among them, the internal tandem duplication (ITD) of this gene is the most common mutation type, accounting for 25-30% of all gene mutation types in AML patients, with a high recurrence rate and poor prognosis (1). Under normal circumstances, the receptor tyrosine kinase FLT3 is expressed on the cell surface of hematopoietic stem/progenitor cells. It binds to its ligand and could be activated with downstream signaling pathway activation such as PI3K/AKT, STATs, and MAPKs, thereby precisely regulating the hematopoiesis process (2). When the FLT3-ITD mutation occurs, the protein no longer depends on ligand binding but remains in an activated state. Meanwhile, combined with the abnormal activation of its downstream signaling pathways, it causes the differentiation of hematopoietic cells and continuous malignant proliferation, ultimately leading to carcinogenesis (2). Therefore, it is particularly important to develop novel therapeutic drugs for FLT3-ITD AML.

The new generation of receptor tyrosine kinase (RTK) FLT3 selective inhibitors, such as quizartinib (AC220), indicate that although these drugs can significantly improve the survival rate of patients with FLT3-ITD AML (3), the tyrosine kinase domain (TKD) of FLT3 often undergoes secondary mutations after treatment, or/and mutations occur in cancer signaling pathways such as RAS/MAPK. The resulting drug resistance greatly limits the long-term efficacy of such tyrosine kinase inhibitors. In addition, even after hematopoietic stem cell transplantation, patients with newly diagnosed FLT3-ITD AML have a higher recurrence rate and a shorter survival time compared with patients with AML without FLT3 mutations (3, 4). Relevant studies have found that the prognoses of different AML patients who are all positive for FLT-ITD are not homogeneous. Even after allogeneic hematopoietic stem cell transplantation, the degrees of benefit vary among different patients (5). We speculate that this may be highly related to the immune escape of cancer cells mediated by the immune system regulators, mainly the immune checkpoint (IC) (6). Usually, immune regulatory pathways such as PD-L1/PD-1 can be “hijacked” by cancer cells and continuously activated, suppressing anti-cancer immunity and thus promoting the occurrence of cancer. As one of the fatal diseases, cancer malignancy is not only because of its infinite proliferation, but also because of its cunning “immune escape” ability. In the human immune system, T cells are the “main force” in identifying and eliminating abnormal cells, but cancer cells have evolved a sophisticated “braking system”, the PD-1/PD-L1 signaling axis, which achieves self-preservation by inhibiting T cell activity. Significant breakthroughs have been made in cancer immunotherapy by using monoclonal antibodies targeting programmed death receptor 1 (PD-1), or programmed death ligand 1 (PD-L1) for checkpoint blockade (7–9). However, despite the advantages of such kind of immunotherapies, it has only been successful in a small subset of patients.

In the present study, we discovered that the spleen tyrosine kinase (SYK) inhibitor fostamatinib, an orally available small molecule to treat chronic immune thrombocytopenia (ITP), displayed drug repurposing with potential ability to inhibit FLT3-ITD+ AML cell proliferation and cause leukemic cell apoptosis. Network pharmacology analyses further revealed the potential pharmacological mechanism associated with PI3K-AKT signaling. Additionally, the immune checkpoints including PD-L1 and CD47 were also down regulated by fostamatinib treatment. In sum, this study provided a proof of concept to evaluate the benefits of drug repurposing in AML drug development.

## Materials and methods

### Cells, reagents, drugs and instruments

The cells used in this experiment were the FLT3-ITD+ AML cell line MV4-11 (purchased from Procell, Wuhan, China). The cells were cultured in an incubator at 37°C with 5% CO_2_, and the fresh culture medium was replaced every two days. The culture medium was RPMI-1640 medium produced by Thermo Fisher Scientific, USA (with an additional 1% penicillin-streptomycin and 10% fetal bovine serum). All the tested drugs were purchased from Selleck, USA, with a storage concentration of 10 mM, dissolved in DMSO and stored in a -20°C refrigerator. The reagent used for the cell viability assay was the CellTiter-Glo^®^ Luminescent Cell Viability Assay Kit (G7571, Promega, USA), and the reagent used for the cell apoptosis assay was the Caspase 3/7 Activity Cell Apoptosis Assay Kit (C10432, Thermo Fisher Scientific, USA). The instruments involved in the cytological experiments were the iD5 Multimode Microplate Reader (ELISA reader) (Molecular Device, USA) for detecting cell proliferation and viability, and the FACSAria Flow Cytometry Sorting Instrument (BD, USA) for detecting cell apoptosis.

### Cytological experimental methods

#### Cell proliferation assay

After counting the suspension of MV4–11 cells in normal growth state, they were plated in 96-well cell culture plates, with 5000 cells/100 μl per well. Different drug concentration gradients and a DMSO control were set respectively, and then the plates were cultured in an incubator at 37°C with 5% CO_2_ for 48 h. Then, according to the instructions of the CellTiter-Glo^®^ Luminescent Cell Viability Assay Kit (G7571, Promega, USA), after adding the corresponding detection reagent, the cell proliferation and viability were detected by the iD5 Multimode Microplate Reader (ELISA reader).

#### Cell apoptosis assay

Different drug concentrations were added to the suspension of MV4–11 cells in normal growth state. After culturing for a period of time, the cells were collected by centrifugation at 1500 rpm. Then, according to the instructions of the Caspase 3/7 Activity Cell Apoptosis Assay Kit (C10432, Thermo Fisher Scientific, USA), after adding the detection reagent, the cell apoptosis was detected and analyzed by the FACSAria Flow Cytometry Sorting Instrument (BD, USA).

#### Cell surface immune checkpoint expression detection

Different drug concentrations were added to the suspension of MV4–11 cells in culture. After culturing for a period of time, the cells were collected by centrifugation at 1500 rpm. The cells were blocked in 0.5% BSA-PBS solution for half an hour and then incubated using the relevant primary antibody. After washing for 3 times, the fluorescent secondary antibody was used for incubation. Isotype control samples were set accordingly. After washing, the cells were collected for detection by the FACSAria Flow Cytometry Sorting Instrument (BD, USA).

### Network pharmacology experimental methods

#### Query and screening of drug and disease targets

First, the 3D structure of the candidate drug was searched in the Pubchem database (http://pubchem.ncbi.nlm.nih.gov/), and their 3D structure file was imported into the PharmMapper database (http://lilab-ecust.cn/pharmmapper/submitfile.html) to screen for the possible targets of the drugs. Then, the possible targets related to FLT3-ITD AML disease were further searched in the GeneCards database (http://www.genecards.org/). Finally, the Venn online platform (http://bioinformatics.psb.ugent.be/webtools/Venn/) was used to take the intersection of the drug and disease targets for subsequent analysis.

#### Protein-protein interaction network analysis

The network visualization construction of Protein-Protein Interaction (PPI) and the analysis of the String database (https://string-db.org/) is a database for searching the interactions between proteins, aiming to provide the evaluation and integration of PPI. The intersection targets obtained by taking the intersection through the Venn diagram were input into the String database to construct the PPI network diagram.

#### KEGG signaling pathway enrichment and construction of the “drug-target-pathway-disease” network diagram

A complete set of online functional annotation tools, the David database (https://david.ncifcrf.gov/), is available for researchers to understand the biological significance and related signaling pathways behind a large number of genes. The intersection of drug and disease targets was input into the David database for KEGG signaling pathway analysis, and then the related targets could be imported into the Cytoscape software to construct a visual “Drug-Target-Pathway-Disease” network diagram.

### Statistical analysis

The experimental data were analyzed using GraphPad Prism 9.0 statistical software. The statistical graphs of the data were expressed as the mean ± standard error of three independent experiments. The Student’s t-test was used to compare the differences between groups. When P < 0.05, the difference between groups was considered statistically significant.

## Results

### Fostamatinib inhibits FLT3-ITD+ AML cell proliferation and induces cell apoptosis

**Figure 1:**
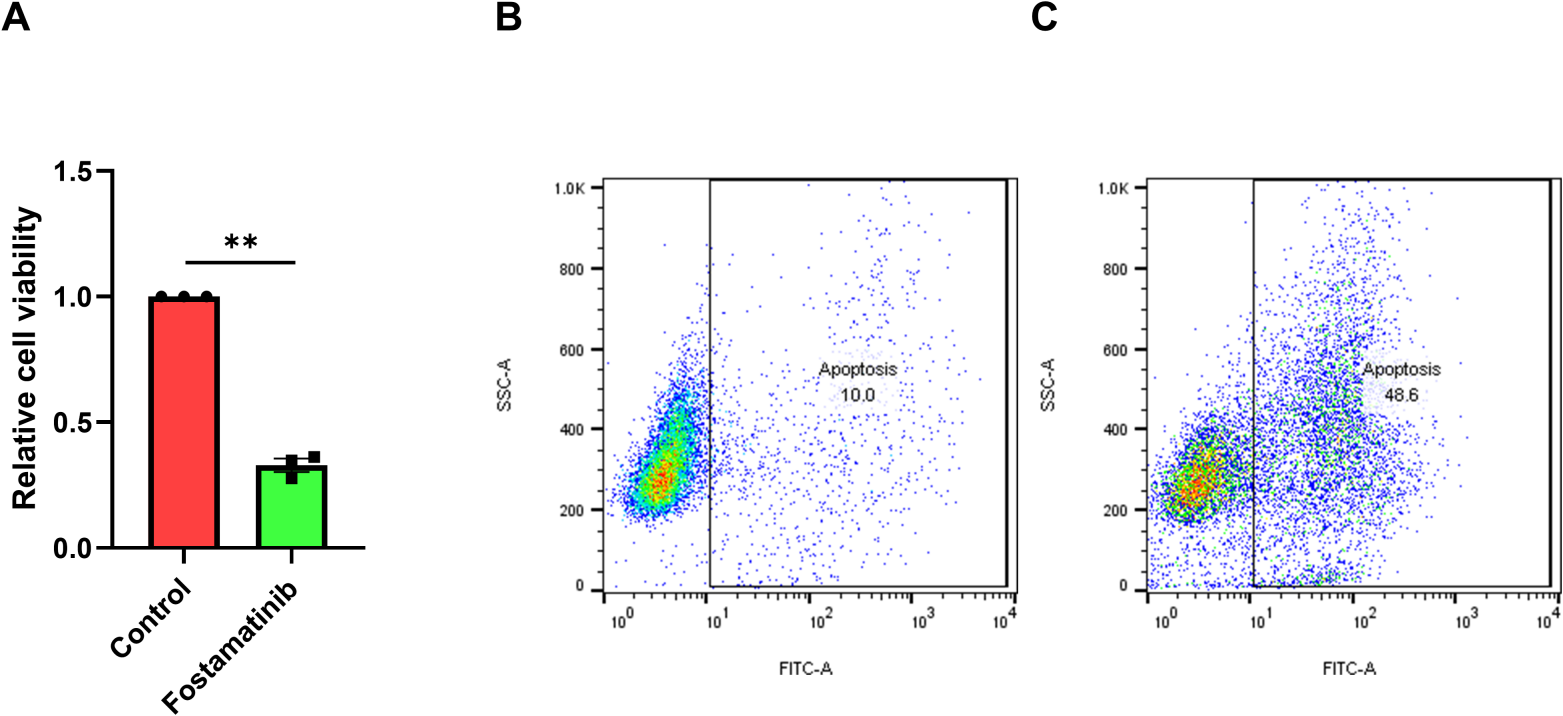
Fostamatinib treatment inhibited cell proliferation and induced cell apoptosis against FLT3-ITD AML. **(A)** Fostamatinib treatment (100 nM for 48 h) could inhibit cell proliferation in MV4–11 cell culture. ** indicated P < 0.01. **(B)** Fostamatinib treatment (250 nM for 48 h) could induce cell apoptosis in MV4–11 cell culture.

To find the novel potential candidate that inhibit AML cell proliferation, we recruited the CellTiter-Glo^®^Luminescent Cell Viability Assay and found fostamatinib inhibited FLT3-ITD+ AML MV4–11 cell proliferation after 100 nM treatment for 48 h (**Figure 1A**). In addition, the flow cytometry analyses indicated that nearly half of these leukemic cells went apoptosis after 250 nM fostamatinib treatment for 48 h by performing Caspase 3/7 Activity Cell Apoptosis Assay using flow cytometry analyses (**Figures 1B, C**). All these above data suggest that fostamatinib inhibited FLT3-ITD+ AML cell proliferation and induced cell apoptosis, which could be deemed as one novel potential drug candidate against FLT3-ITD+ AML.

### Network pharmacology analyses decipher associated pharmacological mechanism

**Figure 2:**
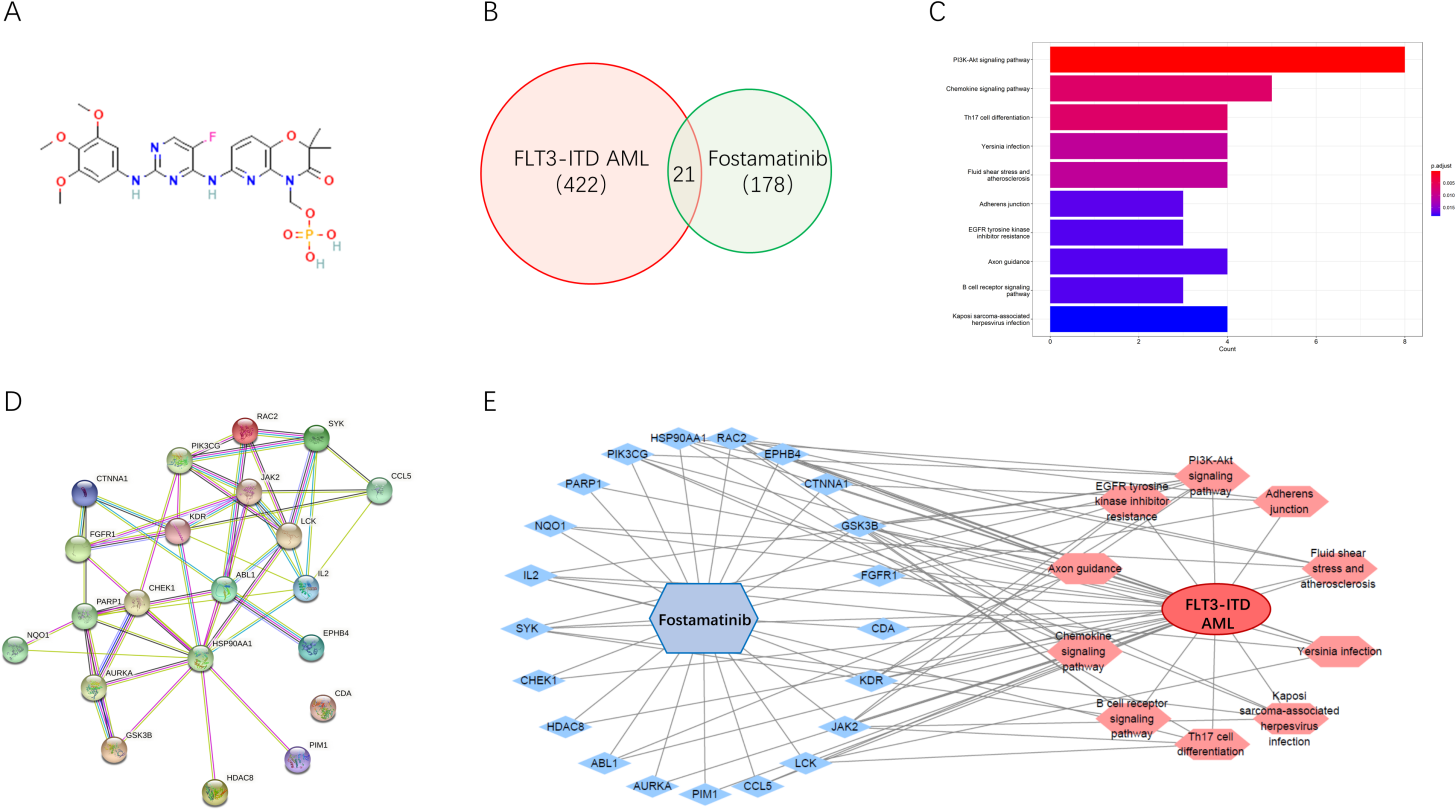
Network pharmacology analyses deciphered associated pharmacological mechanism. **(A)** Chemical structure of fostamatinib. **(B)** Venn map of the common targets. **(C)** KEGG pathway enrichment analysis of the common targets. **(D)** PPI diagram of the targets. **(E)** A visual network diagram of “drug-target-pathway-disease” for fostamatinib against FLT3-ITD AML.

Furthermore, we would take the network pharmacology analyses for potential mechanism exploration. The 3D structure of fostamatinib is shown in **Figure 2A**. After importing it into the PharmMapper database for screening, a total of 178 drug targets were obtained. Then, we searched for 422 targets related to FLT3-ITD AML disease in the GeneCards database. By taking the intersection through the venn online platform, we finally obtained 21 common targets (**Figure 2B**). For these potential targets, many interesting signaling pathways were screened out through the KEGG pathway enrichment analysis using David (**Figure 2C**). The visualization results showed that cancer associated signaling pathways, such as the PI3K-AKT signaling pathway, were all enriched. By inputting the information of the intersection targets into the STRING database, we could obtain the PPI diagram of the targets (**Figure 2D**). The network diagram showed that SYK, HSP90AA1, JAK2, AURKA, etc., were all important targets among them. Further, through the relationship between the drug and proteins, a visual network diagram of “drug-target-pathway-disease” was constructed (**Figure 2E**). This indicates that the drug of fostamatinib affects these cancer-related signaling pathways through these related potential targets in the treatment of FLT3-ITD+ AML.

### Fostamatinib downregulates the expressions of immune checkpoints

**Figure 3:**
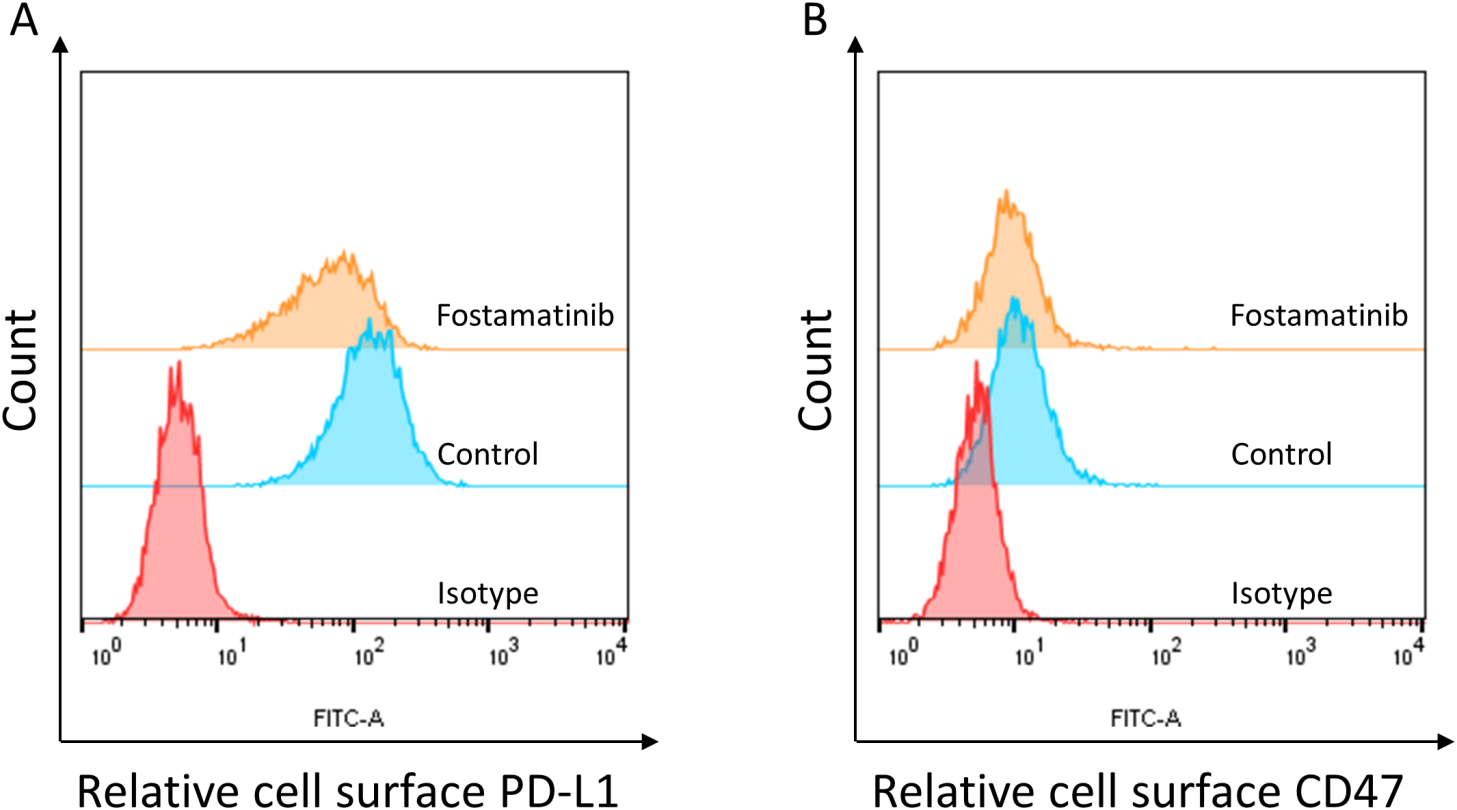
Fostamatinib treatment decreased the cell surface immune checkpoint expression in FLT3-ITD AML cells. **(A)** Fostamatinib treatment (250 nM for 48 h) could obviously decrease the cell surface PD-L1 level in MV4–11 cells. **(B)** Fostamatinib treatment (250 nM for 48 h) could obviously decrease the cell surface CD47 level in MV4–11 cells.

Immune checkpoint expression was associated with many signaling pathways. In the MV4–11 cell model, we found that the cell surface PD-L1 was obviously detected (**Figure 3A**). Interestingly, we found that 250 nM fostamatinib treatment for 48 h could obviously decrease the cell surface PD-L1 level in MV4–11 cells (**Figure 3A**). In addition, we also found that the cell surface CD47 was obviously detected (**Figure 3B**) and 250 nM fostamatinib treatment for 48 h could also obviously decrease the cell surface CD47 level in MV4–11 cells (**Figure 3B**). Taken together, these data indicated that fostamatinib downregulated the expressions of immune checkpoints including PD-L1 and CD47 at least in the tested MV4–11 AML cells.

## Discussion

AML is a malignant hematological disease caused by lesions of myeloid hematopoietic stem/progenitor cells. With a relatively low survival rate of patients, AML is currently the most common type of acute leukemia in adults (10). In clinical treatment, the traditional “7 + 3” induction intensive chemotherapy regimen (a combination of cytarabine and anthracycline drugs) has always been the mainstay. There has been no revolutionary breakthrough for decades. Although some patients can be cured by receiving intensive chemotherapy and/or hematopoietic stem cell transplantation, most AML patients eventually die due to disease recurrence.

Cancer cell often hijacks immune cell for the immune escape via PD-1/PD-L1 signaling axis by inhibiting T cell activity. Immunotherapy has had a widespread impact on the survival of cancer patients. The emergence of immunotherapy as a new pillar of cancer treatment is partially due to the application of immune checkpoint blockade (ICB) drugs in clinical treatment. However, despite the advantages of immunotherapy, it has only been successful in a small subset of patients and these relevant used monoclonal antibodies have many inherent disadvantages including poor oral bioavailability, prolonged tissue retention and half-life time, poor membrane permeability, transportation and storage as well as high cost (11, 12). Thus, small molecule drugs such as PD-1/PD-L1 chemical inhibitors to avoid the drawbacks of therapeutic antibodies were attractively developed.

SYK is a cytosolic non-receptor protein tyrosine kinase and is mainly expressed in hematopoietic cells (13, 14). SYK inhibitor fostamatinib is an orally available small molecule inhibitor of spleen tyrosine kinase that is used to treat chronic immune thrombocytopenia (ITP) (15). In addition, it was also well developed for the treatment of ITP, autoimmune haemolytic anaemia and IgA nephropathy (15). Given the high attrition in new drug discovery and development, along with substantial costs and slow progress, repurposing old drugs for common and rare diseases has emerged as an appealing option. It capitalizes on de-risked compounds, potentially reducing overall development costs and shortening timelines (16). Given the high attrition in new drug discovery and development, along with substantial costs and slow progress, repurposing old drugs for common and rare diseases has emerged as an appealing option. It capitalizes on de-risked compounds, potentially reducing overall development costs and shortening timelines (16). For example, FDA-approved SYK inhibitor, fostamatinib (R788), holds promise as a repurposed drug for ovarian cancer by inhibiting cell proliferation and inducing apoptosis, potentially providing a novel strategy to enhance patient outcomes (17). As a prodrug of the active metabolite R406, fostamatinib is a SYK inhibitor with a half-maximal inhibitory concentration (IC50) of 41 nM. The active metabolite of fostamatinib is mainly bound to plasma proteins. R788 potently inhibits SYK without affecting Lyn protein and has an effect on FLT3 that is five-fold lower compared to that on SYK. As a prodrug of the SYK inhibitor R406, R788 binds to adenosine triphosphate (ATP) in a competitive manner, with an inhibition constant (Ki) of 30 nM. In human mast cells, macrophages, and neutrophils, R788 specifically inhibits the FcγR signaling pathway. Additionally, R788 can suppress local inflammatory damage mediated by immune complexes (18). Studies have shown that R788 can induce apoptosis in most of the tested diffuse large B-cell lymphoma (DLBCL) cell lines. In R788-sensitive DLBCL cell lines, R788 specifically inhibits the B-cell receptor (BCR) signaling pathway induced by stimuli and ligands, including autophosphorylation at the SYK525/526 sites and SYK-dependent phosphorylation of the B-cell linker protein (BLNK) (19). SYK inhibition could also reprogram tumor immune microenvironment, re-educated protumorigenic macrophages towards an immunostimulatory phenotype in pancreatic ductal adenocarcinoma (20). Inhibitors of SYK are emerging as an exciting new class of agents for treating common B-cell malignancies (14). The heterogeneity of patient-derived AML cells was also examined, and a subgroup of patients underwent *in vitro* SYK inhibition (21). However, larger studies are urgently required to ascertain which patient subgroups would benefit most from this treatment (21). Fostamatinib has an effect on FLT3 that is five-fold lower compared to that on SYK. FLT3-ITD is the main therapeutic target in AML. First, fostamatinib will have certain effect on FLT3-ITD, which disturbed the down-stream oncogenic signaling pathways like AKT, MAPK. etc. Second, the target of Syk is also reported to be associated with AML development. For example, proteomic and genetic approaches identify SYK as an AML target (22). Thus, fostamatinib could also target directly SYK as a potential AML drug. Taken together, fostamatinib targeted SYK and FLT3-ITD, which are both therapeutic targets in AML.

**Figure 4:**
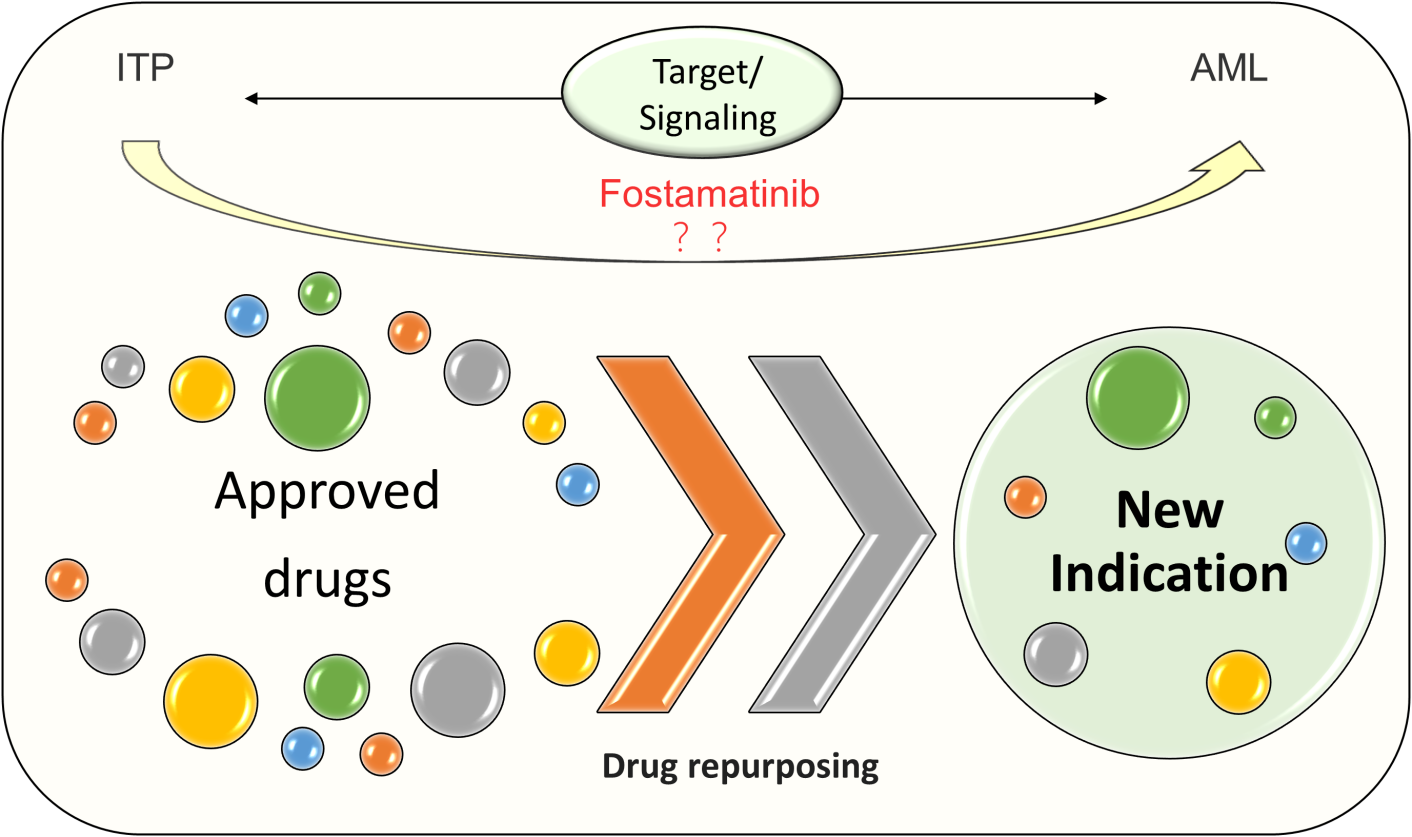
Hypothesis of drug repurposing concerning the common targets/signalings in different diseases.

Targeted therapies in cancer aim to inhibit specific molecular targets responsible for enhanced tumor growth. AKT/PKB (protein kinase B) is a serine threonine kinase involved in several critical cellular pathways, including survival, proliferation, invasion, apoptosis (23). Cancer treatment by chemotherapy and gamma-irradiation kills target cells primarily by the induction of apoptosis. The PI3K/AKT pathway is involved in many of the mechanisms targeted by these new drugs (23, 24). Thus, consistent to the previous studies, our present study indicated that AKT is the main pharmacological signaling, which caused AML cancer cell apoptosis. Fostamatinib induced significant apoptosis in primary AML cells, although the proapoptotic effects of the Syk inhibitors were less pronounced than the antiproliferative effects, which concluded that the Syk inhibitors had antileukemic effects in AML (21). Proteomic and genetic approaches identify SYK as an AML target (22). Thus, Syk is an important oncogene and signaling mediator activated by cell surface receptors crucial for AML maintenance and progression. SYK inhibition targets and kills AML stem cells by blocking their oxidative metabolism (25). These previous results identify gene expression profile signatures that may predict sensitivity to SYK inhibition and underscore the potential for personalized therapeutic strategies in AML (26). The cytoplasmic SYK has emerged as a promising therapeutic target in AML due to its role in promoting leukemic cell survival, proliferation, and chemoresistance. Taken together, the previous studies and our present study consistently indicated that fostamatinib is a potential AML drug as drug repurposing manner. Immune checkpoint regulation is associated with many transcriptional factors. For example, MYC regulates the antitumor immune response through CD47 and PD-L1 (27). Moreover, c-MYC oncogenic network promotes the maintenance and drug resistance of human FLT3-ITD AML stem cells (28). It is proved that FLT3-AKT and its downstream targets GSK3β, c-Myc, and cyclin D1, cooperatively up-regulate the pro-apoptosis proteins Bim and Bax, and down-regulate the anti-apoptosis protein Mcl1. In FLT3-ITD positive AML, targeting FLT3-AKT-c-Myc pathway is a promising treatment strategy for patients with FLT3-ITD+ AML (29). Therefore, FLT3-AKT-c-Myc pathway is a potential mechanism of immune checkpoint regulation and fostamatinib affects the immune checkpoint regulation, which might be via the FLT3-AKT-c-Myc pathway. The conceptual basis was shown taking fostamatinib as a promising candidate against AML for drug repurposing (**Figure 4**). Consistently, in the present study, we found fostamatinib showed the anti-cancer effects in FLT3 mutated AML.

Taken together, we found that fostamatinib exhibited drug repurposing potential by inhibiting the proliferation of FLT3-ITD+ AML cells, inducing leukemic cell apoptosis and reducing immune checkpoint expressions. Specifically, it evaluated advantages in terms of cytotoxicity and small - molecule immune checkpoint inhibitors. This study established a conceptual framework for assessing the benefits of drug repurposing in AML novel drug development.

## Data Availability

The raw data supporting the conclusions of this article will be made available by the authors, without undue reservation.

## References

[1] Abu-duhier FM Goodeve AC Wilson GA Gari MA Peake IR Rees DC. FLT3 internal tandem duplication mutations in adult acute myeloid leukaemia define a high-risk group. Br J haematology. (2000) 111:190–5. doi: 10.1046/j.1365-2141.2000.02317.x 11091200

[2] Schmidt-arras D Bohmer SA Koch S Müller JP Blei L Cornils H. Anchoring of FLT3 in the endoplasmic reticulum alters signaling quality. Blood. (2009) 113:3568–76. doi: 10.1182/blood-2007-10-121426 19204327

[3] Thomas CM Campbell P. FLT3 inhibitors in acute myeloid leukemia: Current and future. J Oncol Pharm Pract. (2019) 25:163–71. doi: 10.1177/1078155218802620 30270754

[4] Schmid C Labopin M Socie G Daguindau E Volin L Huynh A. Outcome of patients with distinct molecular genotypes and cytogenetically normal AML after allogeneic transplantation. Blood. (2015) 126:2062–9. doi: 10.1182/blood-2015-06-651562 26351297

[5] Schlenk RF Kayser S Bullinger L Held G Lübbert M Kindler T. Differential impact of allelic ratio and insertion site in FLT3-ITD-positive AML with respect to allogeneic transplantation. Blood. (2014) 124:3441–9. doi: 10.1182/blood-2014-05-578070 25270908

[6] Sharma P Allison JP. The future of immune checkpoint therapy. Science. (2015) 348:56–61. doi: 10.1126/science.aaa8172 25838373

[7] Inman BA Longo TA Ramalingam S Harrison MR. Atezolizumab: A PD-L1-blocking antibody for bladder cancer. Clin Cancer Res. (2017) 23:1886–90. doi: 10.1158/1078-0432.CCR-16-1417 27903674

[8] Powles T O’Donnell PH Massard C Arkenau HT Friedlander TW Hoimes CJ. Efficacy and safety of durvalumab in locally advanced or metastatic urothelial carcinoma: updated results from a phase 1/2 open-label study. JAMA Oncol. (2017) 3:e172411. doi: 10.1001/jamaoncol.2017.2411 28817753 PMC5824288

[9] Robert C Schachter J Long GV Arance A Grob JJ Mortier L. Pembrolizumab versus ipilimumab in advanced melanoma. N Engl J Med. (2015) 372:2521–32. doi: 10.1056/NEJMoa1503093 25891173

[10] Newell LF Cook RJ. Advances in acute myeloid leukemia. BMJ. (2021) 375:n2026. doi: 10.1136/bmj.n2026 34615640

[11] Naidoo J Page DB Li BT Connell LC Schindler K Lacouture ME. Toxicities of the anti-PD-1 and anti-PD-L1 immune checkpoint antibodies. Ann Oncol. (2015) 26:2375–91. doi: 10.1093/annonc/mdv383 PMC6267867 26371282

[12] Boussiotis VA. Molecular and biochemical aspects of the PD-1 checkpoint pathway. N Engl J Med. (2016) 375:1767–78. doi: 10.1056/NEJMra1514296 PMC5575761 27806234

[13] Liu D Mamorska-Dyga A. Syk inhibitors in clinical development for hematological Malignancies. J Hematol Oncol. (2017) 10:145. doi: 10.1186/s13045-017-0512-1 28754125 PMC5534090

[14] Efremov D Laurenti L. The Syk kinase as a therapeutic target in leukemia and lymphoma. Expert Opin Investig Drugs. (2011) 20:623–36. doi: 10.1517/13543784.2011.570329 21438742

[15] Markham A. Fostamatinib: first global approval. Drugs. (2018) 78:959–63. doi: 10.1007/s40265-018-0927-1 29869203

[16] Pushpakom S Iorio F Eyers PA Escott KJ Hopper S Wells A. Drug repurposing: progress, challenges and recommendations. Nat Rev Drug Discovery. (2019) 18:41–58. doi: 10.1038/nrd.2018.168 30310233

[17] Lee HM Cho HJ Lee YM Kim HJ Heo K. Fostamatinib inhibits the proliferation of ovarian cancer cells through apoptosis induction. Anticancer Res. (2024) 44:4895–903. doi: 10.21873/anticanres.17315 39477304

[18] Braselmann S Taylor V Zhao H Wang S Sylvain C Baluom M. R406, an orally available spleen tyrosine kinase inhibitor blocks fc receptor signaling and reduces immune complex-mediated inflammation. J Pharmacol Exp Ther. (2006) 319:998–1008. doi: 10.1124/jpet.106.109058 16946104

[19] Chen L Monti S Juszczynski P Daley J Chen W Witzig TE. SYK-dependent tonic B-cell receptor signaling is a rational treatment target in diffuse large B-cell lymphoma. Blood. (2008) 111:2230–7. doi: 10.1182/blood-2007-07-100115 PMC2234057 18006696

[20] Rohila D Park IH Pham TV Weitz J Mendoza TH Madheswaran S. Syk inhibition reprograms tumor-associated macrophages and overcomes gemcitabine-induced immunosuppression in pancreatic ductal adenocarcinoma. Cancer Res. (2023) 83:2675–89. doi: 10.1158/0008-5472.CAN-22-3645 PMC10416758 37306759

[21] Brattås MK Hemsing AL Rye KP Hatfield KJ Reikvam H. Heterogeneity of patient-derived acute myeloid leukemia cells subjected to SYK *in vitro* inhibition. Int J Mol Sci. (2022) 23:14706. doi: 10.3390/ijms232314706 36499034 PMC9737311

[22] Hahn CK Berchuck JE Ross KN Kakoza RM Clauser K Schinzel AC. Proteomic and genetic approaches identify Syk as an AML target. Cancer Cell. (2009) 16:281–94. doi: 10.1016/j.ccr.2009.08.018 PMC2803063 19800574

[23] Shariati M Meric-Bernstam F. Targeting AKT for cancer therapy. Expert Opin Investig Drugs. (2019) 28:977–88. doi: 10.1080/13543784.2019.1676726 PMC6901085 31594388

[24] Vara J Casado E Castro J Cejas P Iniesta CB Barón MG. PI3K/Akt signalling pathway and cancer. Cancer Treat Rev. (2004) 30:193–204. doi: 10.1016/j.ctrv.2003.07.007 15023437

[25] Polak A Bialopiotrowicz E Krzymieniewska B Wozniak J Stojak M Cybulska M. SYK inhibition targets acute myeloid leukemia stem cells by blocking their oxidative metabolism. Cell Death Dis. (2020) 11:956. doi: 10.1038/s41419-020-03156-8 33159047 PMC7648638

[26] Brattås MK Görtler F Johansen S Rye KP Hatfield KJ Reikvam H. Gene expression profiling in acute myeloid leukemia patient subgroups with high and low sensitivity toward SYK inhibitors. Hematol Oncol. (2025) 43:e70058. doi: 10.1002/hon.70058 40088478

[27] Casey SC Tong L Li Y Do R Walz S Fitzgerald KN. MYC regulates the antitumor immune response through CD47 and PD-L1. Science. (2016) 352:227–31. doi: 10.1126/science.aac9935 PMC4940030 26966191

[28] Li L Osdal T Ho Y Chun S McDonald T Agarwal P. SIRT1 activation by a c-MYC oncogenic network promotes the maintenance and drug resistance of human FLT3-ITD acute myeloid leukemia stem cells. Cell Stem Cell. (2014) 15:431–46. doi: 10.1016/j.stem.2014.08.001 PMC4305398 25280219

[29] Wang F Huang J Guo T Zheng Y Zhang L Zhang D. Homoharringtonine synergizes with quizartinib in FLT3-ITD acute myeloid leukemia by targeting FLT3-AKT-c-Myc pathway. Biochem Pharmacol. (2021) 188:114538. doi: 10.1016/j.bcp.2021.114538 33831397

